# Newly Designed Organic-Inorganic Nanocomposite Membrane for Simultaneous Cr and Mn Speciation in Waters

**DOI:** 10.3390/gels11030205

**Published:** 2025-03-15

**Authors:** Penka Vasileva, Irina Karadjova

**Affiliations:** Faculty of Chemistry and Pharmacy, University of Sofia “St. Kliment Ohridski”, 1, J. Bourchier Blvd., 1164 Sofia, Bulgaria

**Keywords:** hybrid hydrogel nanocomposite membrane, polyvinyl alcohol, silica, gold nanoparticles, speciation, chromium (III and VI), manganese (II and VII), tap and wastewaters

## Abstract

A sol-gel approach was used to prepare a thin hydrogel membrane based on an organic-inorganic polymer matrix embedded with pre-synthesized gold nanoparticles (AuNPs). The organic polymers utilized were poly(vinyl alcohol) (PVA) and poly(ethylene oxide) 400 (PEO) while tetraethoxysilane (TEOS) served as a precursor for the inorganic silica polymer. AuNPs were synthesized using D-glucose as a reducing agent and starch as a capping agent. A mixture of PVA, PEO, pre-hydrolyzed TEOS, and AuNP dispersions was cast and dried at 50 °C to obtain the hybrid hydrogel membrane. The structure, morphology, and optical properties of the nanocomposite membrane were analyzed using TEM, SEM, XRD, and UV-Vis spectroscopy. The newly designed hybrid hydrogel membrane was utilized as an efficient sorbent for the simultaneous speciation analysis of valence species of chromium and manganese in water samples via solid-phase extraction. This study revealed that Cr(III) and Mn(II) could be simultaneously adsorbed onto the PVA/PEO/SiO_2_/AuNP membrane at pH 9 while Cr(VI) and Mn(VII) remained in solution due to their inability to bind under these conditions. Under optimized parameters, detection limits and relative standard deviations were determined for chromium and manganese species. The developed analytical method was successfully applied for the simultaneous speciation analysis of chromium and manganese in drinking water and wastewater samples.

## 1. Introduction

The toxicological and biological effects of chemical elements are significantly influenced by their specific chemical forms. It is well known that the toxicity of metal ions varies based on their oxidation states, which directly impact their physicochemical behavior, biological interactions, and bioavailability [1]. This highlights the importance of speciation analysis in assessing environmental quality, food safety, and pharmaceutical products [2,3].

Chromium is one of the most abundant elements on Earth, naturally present in rocks, soil, plants, animals, volcanic emissions, and atmospheric gases. In aqueous environments, chromium mainly exists in two oxidation states: Cr(III) and Cr(VI), each exhibiting distinct toxicity, mobility, and bioavailability [4,5,6]. While Cr(III) is an essential micronutrient for living organisms, Cr(VI) is highly toxic and has been linked to carcinogenic effects in humans [7,8]. Although Cr(VI) can occur naturally in surface and groundwater, its presence is largely a result of industrial pollution [9].

Manganese is another element found both naturally and as an environmental contaminant. It originates from natural sources such as mineral deposits and over a hundred different manganese-containing minerals. Human activities, including mining, ore processing, steel and iron production, wastewater discharge, sewage sludge disposal, fungicide application, and fossil fuel combustion, contribute significantly to manganese pollution. As an essential nutrient for humans and animals, manganese is commonly found in surface and groundwater in various forms, including Mn(II), Mn(IV) particulates, and Mn(IV) hydroxide [10]. The primary sources of manganese intake in humans are drinking water and food. However, both manganese deficiency and excessive exposure can lead to adverse health effects. High levels of manganese have been associated with DNA mutations and neurological disorders, such as manganism, liver dysfunction, hallucinations, depression, and excessive drowsiness. Manganese(VII), widely used as an oxidizing agent in synthetic chemistry and water disinfection, often enters the drinking water systems [11,12]. Although the concentration of KMnO_4_ used in water treatment can be controlled, residual KMnO_4_ poses potential health risks, particularly due to its long-term effects [11,12]. Therefore, accurately determining the residual KMnO_4_ concentration in treated water is crucial for ensuring drinking water safety.

Precise determination of Cr(III)/Cr(VI) and Mn(II)/Mn(VII) in different water sources, including surface water, drinking water, and wastewater, is essential for maintaining water quality standards. Several separation techniques, predominantly based on solid-phase extraction, have been developed for chromium [6,13,14,15,16,17,18] and manganese speciation [18,19,20]. However, simultaneous speciation of both elements remains relatively unexplored [21,22,23,24]. The effectiveness of these procedures largely depends on the selection of the sorbent material, making the development of advanced solid sorbents crucial for analytical applications.

In recent years, nanomaterials—often with additional surface modifications—have emerged as efficient sorbents due to their high surface area, strong chemical activity, rapid kinetics, and selective affinity for heavy metal ions [25]. Examples include noble metal (silver, gold, palladium) and metal-oxide-based (TiO_2_, ZrO_2_, ZnO, Al_2_O_3_) nanoparticles [26,27,28], graphene, carbon nanotubes [29], three-dimensional porous aerogels [30], and layered double hydroxides [31]. However, isolating nanosized sorbents is often complex and time consuming, limiting their widespread application in speciation analysis and trace metal detection. Additionally, their use in dynamic extraction processes can be hindered by high column back pressure. To overcome these challenges, nanocomposites incorporating magnetic cores (Fe_3_O_4_) have been developed, allowing for the rapid and selective separation of toxic metal species [32,33,34].

Organic-inorganic hybrid polymeric materials have gained significant attention for removing toxic contaminants from wastewater and for solid-phase extractions in analytical processes [35,36]. These materials combine the advantageous properties of both organic and inorganic components, resulting in enhanced selectivity for metal ions and improved efficiency in water treatment and analytical applications. Various synthesis techniques are used to create these hybrid materials, including sol-gel processing, self-assembly, nanobuilding block integration (such as layered or core-shell structures), and the development of interpenetrating networks with hierarchical architectures. Recently, silica-supported organic-inorganic hybrid sorbents, synthesized through ion imprinting combined with sol-gel processing, have shown superior selectivity and adsorption capacity. These materials help overcome mass transfer limitations and enable efficient heavy metal removal from aqueous solutions [37,38,39]. However, the primary challenge in using organic-inorganic hybrids for metal separation and detection lies in identifying and developing innovative sorbents with enhanced properties.

This research focused on the preparation and characterization of a novel hybrid hydrogel nanocomposite membrane (PVA/PEO/SiO_2_/AuNPs) and its application as an effective sorbent for the simultaneous speciation and determination of the valence species of chromium and manganese in water samples using selective solid-phase extraction. The nanocomposite membrane was prepared using poly(vinyl alcohol) and poly(ethylene oxide) as a film-forming substance and porogen agent, respectively, while silica sol and starch-coated gold nanoparticles act as crosslinking and mechanically stabilizing components. The proposed analytical method is based on the ability of posively charged Mn(II) and Cr(III) species to be quantitatively retained on the negatively charged PVA/PEO/SiO_2_/AuNP membrane at an optimal pH value while Mn(VII) and Cr(VI) oxyanions remain in solution due to electrostatic repulsion at this condition. This approach offers a simple and efficient process, eliminating the need for filtration or centrifugation. The membrane can be easily removed with plastic tweezers, after which the toxic Cr(VI) and Mn(VII) species are analyzed in the remaining solution. The method’s detection limits and analytical precision make it suitable for national monitoring programs.

## 2. Results and Discussion

### 2.1. Characterization of Gold Nanoparticles and the PVA/PEO/SiO_2_/AuNP Nanocomposite Membrane

**Figure 1:**
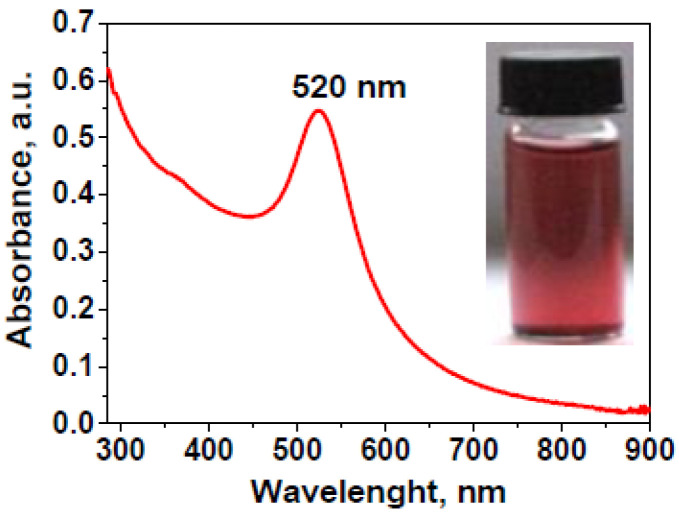
UV-Vis absorption spectrum of as-synthesized starch-coated gold nanoparticles in aqueous dispersion; the inset shows an optical photograph of as-prepared AuNP dispersion.

The optical properties of the gold nanoparticles were studied using UV-Vis spectroscopy and the solid curve in Figure 1 shows the UV-Vis spectrum recorded at 25 °C from the starch-coated AuNPs in wine-red aqueous dipersion, as shown in the inset of Figure 1. The appearance of a single, strong, and sharp plasmon resonance (SPR) band at the wavelength of maximum absorbance λ_max_ = 520 nm indicates the formation of gold particles with nanometer-sized dimensions. Stability was observed for the obtained aqueous dispersion of AuNPs at room temperature for over six months, with no noticeable signs of sedimentation or changes in their UV-Vis absorption spectra. This observation is consistent with the results from measurements of the surface ζ-potential of the synthesized gold nanoparticles: ζ = −28.7 ± 1.6 mV at pH 6.8.

**Figure 2:**
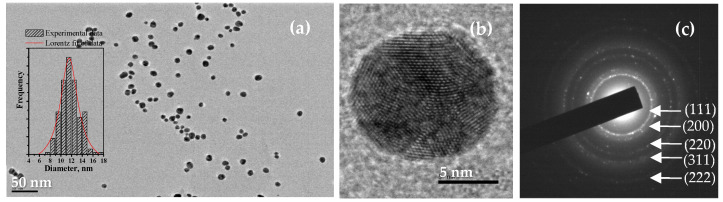
(**a**) TEM and (**b**) HRTEM micrographs of starch-coated gold nanoparticles; (**c**) electron diffraction image; inset in (**a**): nanoparticle size distribution histogram based on measurements of over 200 particles.

The SPR band of starch-coated AuNPs, indicating the formation of uniform spherical nanoparticles, is further confirmed by the TEM and HRTEM micrographs shown in Figure 2 and the corresponding nanoparticle size distribution histogram in the inset of Figure 2. The gold nanoparticles have shapes close to spherical and exhibit a relatively narrow size distribution with an average diameter of 11.8 ± 1.7 nm (Figure 2a). The histogram shows a narrow monomodal size distribution for the synthesized gold nanoparticles. The observed small standard deviation indicates that a reduction with D-glucose in a starch solution with appropriate alkalinity and under the influence of ultrasound leads to the formation of homogeneous nanoparticles in terms of shape and size. In the HRTEM micrograph presented in Figure 2b, the individual crystalline planes in the respective crystal lattices are clearly visible. The electron diffraction image presented in Figure 2c characterizes the synthesized starch-coated gold nanoparticles as a polycrystalline sample.

The nanocrystalline nature of starch-coated AuNPs is further studied by the X-ray diffraction of a layer of as-prepared gold nanoparticles dried at 25 °C (Figure S3). The broad reflection in the 2θ-range of 20–30° originates from the glass substrate of the gold nanoparticle layer subjected to XRD analysis. The low-intensity and broad diffraction peaks at angular positions of 38.2° and 44.5°, corresponding to the (111) and (200) planes of the face-centered cubic (fcc) lattice of metallic gold (PDF 04-0784), indicate the nanocrystallite nature of the metal nanoparticles. (PDF 04-0784).

**Figure 3:**
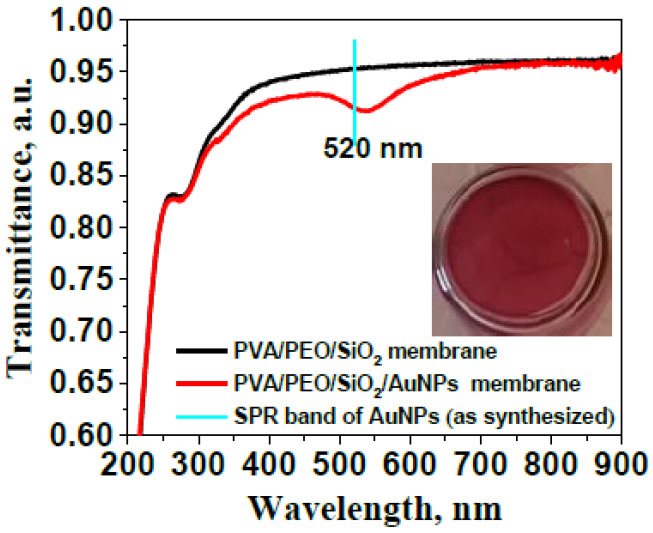
UV-Vis transmission spectra of the PVA/PEO/SiO_2_ hybrid polymer membrane and the membrane modified with AuNPs; the inset shows an optical photograph of the nanocomposite membrane.

The UV-Vis transmission spectrum of the PVA/PEO/SiO_2_/AuNP nanocomposite membrane presented in Figure 3 shows a slight “red” shift of the plasmonic maximum of gold nanoparticles embedded in the hybrid polymer membrane compared to its position in the nanoparticle aqueous dispersion, accompanied by a minor broadening of the plasmon band. This can be interpreted as a result of changes in the dielectric constant of the nanoparticles’ surrounding environment in the hybrid matrix. 

The embedding of AuNPs in a PVA/PEO/SiO_2_ hybrid matrix results in the formation of a transparent, self-standing nanocomposite membrane PVA/PEO/SiO_2_/AuNP with intense and storage-stable coloration (see inset in Figure 3). This is attributed to the homogeneous distribution and stability of gold nanoparticles embedded within the hybrid polymer structure.

**Figure 4:**
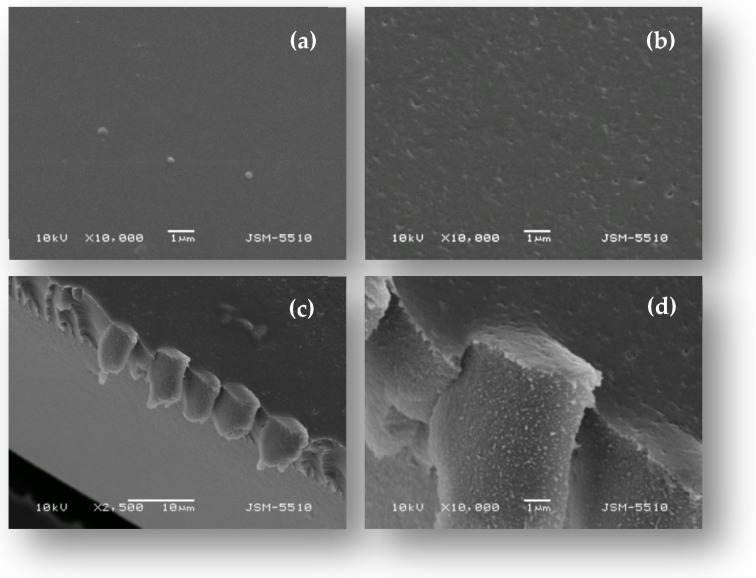
SEM micrographs (top view) of the (**a**) PVA/PEO/SiO_2_hybrid polymer membrane and (**b**) PVA/PEO/SiO_2_/AuNP nanocomposite membranes; (**c**,**d**) SEM micrographs (cross-sections) of the PVA/PEO/SiO_2_/AuNP nanocomposite membrane at different magnifications.

The surface morphology of the PVA/PEO/SiO_2_ hybrid polymer membrane and the membrane modified with starch-coated gold nanoparticles was observed using scanning electron microscopy (SEM) and the obtained results are presented in Figure 4. The PVA/PEO/SiO_2_ hybrid polymer membrane is dense and homogeneous, with a relatively smooth surface. The modification of the hybrid membrane with gold nanoparticles alters the surface morphology of the membrane, which maintains a compact structural integrity but exhibits a less smooth, heterogeneous surface with the presence of surface micropores. To examine the cross-sectional morphology of the nanocomposite membrane using scanning electron microscopy, a membrane sample was immersed in a liquid nitrogen and fractured after solidification. SEM micrographs of the cross-section of the PVA/PEO/SiO_2_/AuNP nanocomposite membrane reveal a dense and microporous structure throughout the membrane’s volume. We believe that the observed folding is an artifact of the sample preparation and is not an inherent structural feature of the nanocomposite membrane.

**Figure 5:**
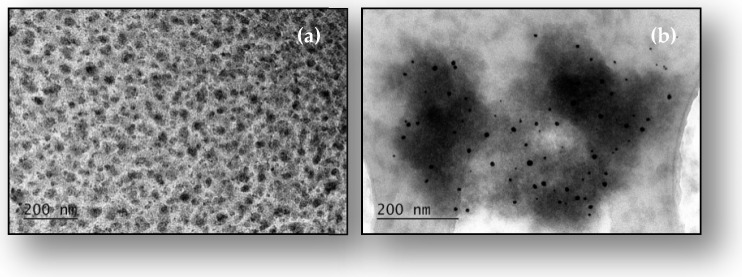
(**a**) and (**b**) TEM micrographs of the PVA/PEO/SiO_2_ hybrid polymer membrane and the membrane modified with AuNPs, respectively.

Electron microscopy observations of the PVA/PEO/SiO_2_ hybrid polymer membrane and the membrane modified with starch-coated AuNPa, obtained directly on a carbon-coated copper grid for TEM observations, are presented in Figure 5a and b, respectively. A relatively homogeneous distribution of AuNPs in the hybrid polymer matrix has been achieved without noticeable changes in the shape and size of the nanoparticles. A dense structure of the PVA/PEO/SiO_2_/AuNP nanocomposite membrane is observed after the incorporation of starch-coated gold nanoparticles in the hybrid polymer matrix. This is a result of additional cross-linking between the starch surface layer of AuNPs and the polymer components of the matrix. The EDX images (Figure S4) corresponding to the TEM image in Figure 5b further indicate preferential cross-linking involving the inorganic silica component of the hybrid microstructure.

It is noteworthy that during handling, the hybrid nanocomposite membrane modified with AuNPs demonstrated superior mechanical strength compared to the PVA/PEO/SiO_2_ hybrid polymer membrane. Furthermore, no leakage of gold nanoparticles was detected from the hydrogel nanocomposite membrane. These experimental results indicate that AuNPs act as an additional cross-linking agent in the formation of the hybrid nanocomposite structure, contributing to enhanced mechanical properties of the membrane.

### 2.2. Adsorption Behavior of the PVA/PEO/SiO_2_/AuNP Nanocomposite Membrane Toward Cr(III)/Cr(VI) and Mn(II)/Mn(VII)—Optimization Studies

The degree of sorption of Cr(III)/Cr(VI) and Mn(II)/Mn(VII) on the PVA/PEO/SiO_2_/AuNP hybrid hydrogel membrane and on the PVA/PEO/SiO_2_ hydrogel membrane was studied in the pH range 3–9 starting with sorption time of 12 h. The amount of gold in the nanocomposite membrane is 596 ± 75 µg, introduced into the Solution 3 (see Section 4.3) when using 5 mL aqueous dispersion of pre-synthesized starch-coated AuNPs [40]. The pH range was chosen based on the assumption that the hybrid polymer matrix, composed of the organic polymer PVA, which contains OH-groups, and the inorganic polymer SiO_2_, which has surface silanol groups (Si-OH), exhibits high sorption activity toward transition metal cations in the pH range 3–9. The results presented in Figure S5 show that quantitative sorption of both Cr(III) and Mn(II) is achieved at pH 9, using the PVA/PEO/SiO_2_/AuNP nanocomposite membrane as a sorbent. Under the same conditions, the PVA/PEO/SiO_2_ membrane without AuNPs did not ensure quantitative sorption for Cr(III) nor for Mn(II). A possible explanation for this experimental finding is the high electrostatic attraction between the negatively charged gold nanoparticles as well as deprotonated free silanol groups (IEP of silica is generally around pH 1.5–3.5) in the nanocomposite membrane and the positively charged Cr(III) and Mn(II) hydroxido complexes that are formed in an ammonia medium at pH 9. This electrostatic attraction facilitates further complex formation with the functional groups of the polymer components in the hybride nanocomposite membrane. In an acidic medium (pH 3), the degrees of sorption for Cr(III) and Mn(II) onto the PVA/PEO/SiO_2_/AuNP hydrogel nanocomposite membrane are lower, approximately 65% and 10%, respectively. The remarcable decrease in Cr(III) and Mn(II) sorption at pH < 7 can likely be attributed to electrostatic repulsion between the positively charged metal complexes—predominant chemical forms of Cr(III) and Mn(II) under these conditions [41]—and the partially protonated, positively charged functional groups of the nanoparticle stabilizing agent (starch), as well as those of the polymer components of the membrane [42]. Additionally, the limited ability of these metal complexes to form further bonds with functional groups due to their inertness further contributes to the reduced sorption.

**Figure 6:**
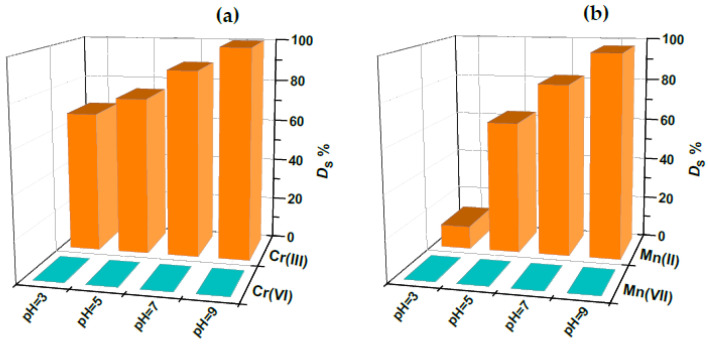
Degree of sorption (*D*_S_, %) of (**a**) Cr(III) in the presence of Cr(VI) and (**b**) Mn(II) in the presence of Mn(VII) onto the PVA/PEO/SiO_2_/AuNP nanocomposite membrane as a function of the pH of the medium; sorption time of 16 h, adsorbent dose (one membrane with the diameter 2.5 cm) of 0.1293 g.

The comparison of the retention behavior of Cr(III)/Cr(VI) and Mn(II)/Mn(VII) is shown in Figure 6a and Figure 6b, respectively, at four different pH values within the range of 3–9. It is seen that the degree of sorption of both Cr(VI) and Mn(VII) is negligible for all studied pH values, mainly due to the electrostatic repulsion between the negative chemical species Cr_2_O_7_^2^⁻ and MnO_4_⁻ and negatively charged gold nanoparticles, as well as negatively charged silanol groups of silica in the membrane. These results clearly demonstrate the potential of the prepared PVA/PEO/SiO_2_/AuNP hybrid nanocomposite membrane for selective and quantitative separation of toxic chromium and manganese species at the defined optimal pH value of 9 and simultaneous speciation of chromium and manganese.

**Figure 7:**
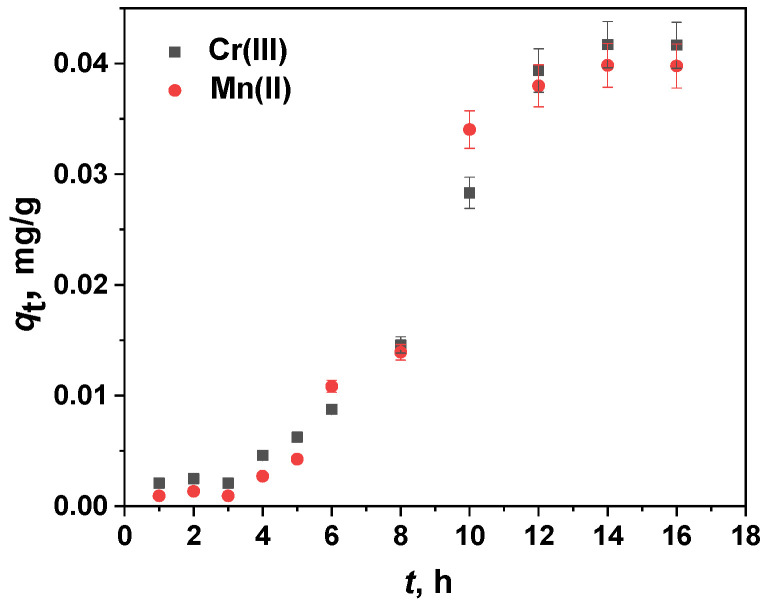
Effect of the contact time on the degree of sorption *D*_s_ of Cr(III) and Mn(II) onto the PVA/PEO/SiO_2_/AuNP nanocomposite membrane at an initial concentration of 1 mg/L (19.23 μmol/L Cr(III) and 18.20 μmol/L Mn(II)), pH of 9, temperature of 25 °C, and adsorbent dose (one membrane with a diameter of 2.5 cm) of 0.1293 g.

In order to optimize the contact time for sorption, the degree of sorption, *D*_S_, of Cr(III) and Mn(II) onto the PVA/PEO/SiO_2_/AuNP membrane sorbent at different contact times was studied in the range 1–16 h. The kinetic adsorption curve is shown in Figure 7 and it can be seen that with an increase in sorption time, the sorbed amounts of both Cr(III) and Mn(II) gradually increase, with quantitative sorption (>99%) being achieved within 12 h. The contact time considered optimal was 12 h. During the solid-phase extraction of the studied ions, the PVA/PEO/SiO_2_/AuNP nanocomposite membrane maintains its integrity and the removal and transfer of the membrane from the vessel is very easy, ensuring the possibility of the simultaneous and selective separation of Cr and Mn species.

A similar relatively slow process (equilibrium time of 12–18 h) has already been reported for the quantitative sorption of Cd(II), Cu(II), Ni(II), Pb(II), and Hg(II) using the L-cysteine-modified chitosan membrane [43]. It is reasonable to assume that such a slow reaching of the adsorption equilibrium is due to the large diffusion barrier in the thin hydrogel membrane. The greater diffusion resistance leads both to the difficult entry of Cr(III) and Mn(II) species into the membrane pores and to their limited association with the sorption centers.

**Figure 8:**
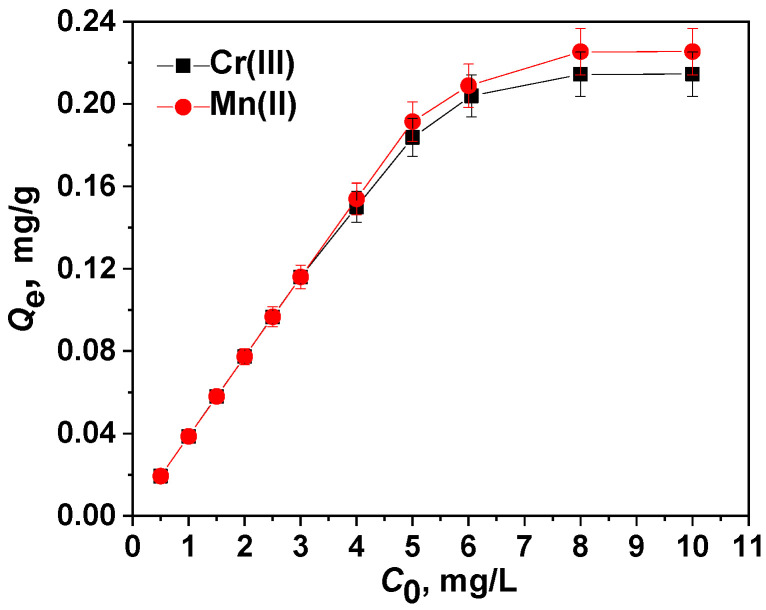
Experimental adsorption isotherms for Cr(III) and Mn(II) sorption on the PVA/PEO/SiO_2_/AuNP nanocomposite membrane (optimal conditions: pH of 9, contact time of 12 h, temperature of 25 °C, adsorbent dose (one membrane with diameter 2.5 cm) of 0.1293 g). The data presented are averaged from three independent experiments.

Under defined optimal conditions, the sorption capacity of the PVA/PEO/SiO_2_/AuNP nanocomposite membrane sorbent was evaluated after the saturation of the membrane with Cr(III) or Mn(II) ions. The effect of the initial concentration of Cr(III) ions or Mn(II) ions (0.5–10 mg/L) on the sorption capacity of the PVA/PEO/SiO_2_/AuNP membrane is displayed in Figure 8.

The adsorption isotherms clearly show that the amount of adsorbed Cr(III) or Mn(II) per unit mass of the membrane increases with growing Cr(III) or Mn(II) concentration and reaches a plateau determining the maximum adsorption capacity (*Q*_e,max_)—0.2144 mg/g for Cr(III) and 0.2254 mg/g for Mn(II).

### 2.3. Desorption Studies

The choice of eluent is very important as the eluting agent must ensure complete elution of Cr(III) and Mn(II) from the nanosorbent—the PVA/PEO/SiO_2_/AuNP membrane. Experiments were conducted with different concentrations of nitric acid: 2 mol/L, 4 mol/L, and conc. HNO_3_ at room temperature. The degree of elution increases with the increase in the concentration of nitric acid, with the maximum value reaching 75% when using conc. HNO_3_. In addition, it was observed that elution at room temperature, regardless of the concentration of nitric acid, leads to the partial but not complete dissolution of the membrane. This provides further evidence that the gold nanoparticles embedded in the hybrid structure of the membrane also act as a crosslinking agent. The complete dissolution of the membrane and, in this way, quantitative recovery of retained elements was achieved with 1 mL of aqua regia under mild heating (~ 40 °C) for 15 min.

### 2.4. Investigations on the Mechanism of Cr(III) and Mn(II) Adsorption onto the PVA/PEO/SiO_2_/AuNP Nanocomposite Membrane

#### 2.4.1. Adsorption Isotherm Models

The experimental data for the adsorption of Cr(III) and Mn(II) ions onto the PVA/PEO/SiO_2_/AuNP hydrogel nanocomposite membrane were analyzed as a function of the initial ion concentrations using four well-established two-parameter adsorption models [44,45,46]: Langmuir (Equation (1)), Freundlich (Equation (2)), Dubinin–Kaganer–Radushkevich (DKR) (Equation (3)), and Temkin (Equation (4)).

The Langmuir isotherm model assumes a monolayer adsorption occurring in a surface monolayer of uniform sites. The linear form of the Langmuir isotherm is presented by Equation (1):

Langmuir: * C*_e_/*Q*_e_ = *C*_e_/*Q*_m_ + 1/(*Q*_m_ × *K*_L_) (1)

where *Q*_e_ (mg/g) is the equilibrium capacity of the membrane, *Q*_m_ (mg/g) is the calculated maximum adsorption capacity, and *K*_L_ (L/mg) is the equilibrium constant of the adsorbate–adsorbent equilibrium.

To predict the favorability of a given adsorption system, it is recommended to use the dimensionless separation factor *R*_L_, which is represented by Equation (2). The isotherm is irreversible, favorable, linear, or unfavorable if *R*_L_ = 0, 0 < *R*_L_ < 1, *R*_L_ = 1, or *R*_L_ > 1, respectively:

*R*_L_ = 1/(1 + *K*_L_ × *C*_0_) (2)

where *C*_0_ (mg/L) is the initial concentration of sorbate in the liquid phase.

The Freundlich isotherm model is not restricted to the monolayer formation and can be applied in the case of multilayer adsorption of the adsorbate on a heterogeneous surface. Equation (3) presents the Freundlich isotherm in the linear form:

Freundlich:  ln *Q*_e_ = ln *K_F_* + (1/*n)* × ln *C_e_* (3)

where *C*_e_ (mg/L) and *Q*_e_ (mg/g) are the Cr(III) or Mn(II) equilibrium concentration in the solution and equilibrium capacity of the membrane, respectively; *K*_F_ is the Freundlich isotherm constant; and *n* is the adsorption intensity. The value of *n* gives information about the adsorbent–adsorbate interaction. The adsorption process is favorable when 0 < 1/*n* < 1; unfavorable when 1/*n* > 1; and irreversible when 1/*n* = 1.

The DKR isotherm model assumes a multilayer adsorption onto micropore heterogeneous surfaces:

DKR:  ln *Q*_e_ = ln *X*_m_ – *β × ε*^2^ (4)

where *Q*_e_ is sorbed amount (mg/g); *X*_m_ is the DKR monolayer capacity (mg/g); *β* is the coefficient associated with the average sorption energy (mol^2^/J^2^); and *ε* is the Polanyi potential, which is equal to:

*ε* = *R × T* × ln(1 + 1/*C*_e_) (5)

where *R* is the gas constant (J/(K×mol); *T* is the absolute temperature (K); and *C*_e_ is the equilibrium concentration of the sorbate (mg/L).

The value of *β* is related to the adsorption energy *E* by the following relationship:

*E* = 1/(2 × *β*)^½^ (6)

The adsorption energy value can be used to determine whether the adsorption process is physical or chemical [47]. When the *E* value is between 8 and 16 kJ/mol, the predominant adsorption process is chemisorption by an ion-exchange mechanism [48].

The Temkin isotherm model is based on the multilayered chemisorption process and this isotherm is applicable to a limited range of ion concentrations:

Temkin:  *Q*_e_ = (*R × T*/*b*) × ln *K_T_* + (*R × T*/*b*) × ln *C_e_* (7)

where *R* is the constant (8.314 J/(mol × K), *T* is the absolute temperature (K), *K*_T_ is the equilibrium binding constant (L/g), and *b* is a constant related to the heat of adsorption *B* = *R × T*/*b* (J/mol).

The final calculated adsorption isotherm parameters are summarized in Table 1, with graphical representations for Cr(III) and Mn(II) shown in Figures S6 and S7, respectively. The suitability of different adsorption models for describing the adsorption behavior of the two analytes on the hybrid hydrogel nanocomposite membrane was evaluated by comparing the correlation coefficient (*R*^2^) values. As shown in Table 1, the Langmuir model exhibited the highest coefficient of determination (*R*^2^ = 0.9998 for Cr(III) and *R*^2^ = 0.9999 for Mn(II)), outperforming the other isotherm models.

The calculated adsorption capacity (*Q*_m_) values closely matched the experimentally obtained values for both ions (*Q*_e,max_ = 0.2144 mg/g for Cr(III) and *Q*_e,max_ = 0.2254 mg/g for Mn(II)), supporting the assumption that adsorption occurs as a monolayer on a homogeneous surface. Additionally, the Langmuir dimensionless factor (*R*_L_) values fell within the range of 0 < *R*_L_ < 1, indicating that the adsorption of Cr(III) and Mn(II) onto the PVA/PEO/SiO_2_/AuNP nanocomposite membrane is favorable. This conclusion is further supported by the Freundlich coefficient (*n*), which satisfies the condition 0 < 1/*n* < 1, confirming favorable adsorption. Moreover, the positive values of adsorption energy (*E*) from the Dubinin–Radushkevich (DKR) isotherm model suggest that the adsorption of Cr(III) and Mn(II) onto the nanocomposite membrane is endothermic [49]. The obtained *E* values were less than 8 kJ/mol, suggesting that the sorption process is mainly driven by physical adsorption.

#### 2.4.2. Modeling of Cr(III) Sorption Kinetics

In order to understand the behavior of Cr(III) and Mn(II) ions adsorbed by the novel PVA/PEO/SiO_2_/AuNP nanocomposite membrane and to determine the controlling mechanism of the adsorption process, the two most commonly used kinetic models (pseudo-first-order and pseudo-second-order) were tested, both of which contain two adjustable parameters [50]. Additionally, two sigmoidal models were considered, as suggested by the S-shaped behavior of the experimental kinetic curves—slogistic1 [51] and dose response [52], which contain three and four adjustable parameters, respectively. All models in their nonlinear form were fitted to the experimental data using the OriginPro 2019 software and an iterative procedure following the Levenberg–Marquardt algorithm:

(8) $$pseudo\text{-}first\text{-}order\;\;q_{t}=q_{e}(1-e^{-k_{1}.t})$$

where *q*_t_ and *q*_e_ (mg/g) are the adsorbed amounts at different times *t* (h) and at an equilibrium, respectively, and *k*_1_ (1/h) is the rate constant. The pseudo-first-order kinetic model better describes an adsorption process controlled by diffusion and is mainly used to simulate a simple single reaction:

(9) $$pseudo\text{-}second\text{-}order\;\;q_{t}=k_{2\;}.q_{e}^{2}\frac{t}{1+k_{2}{.q}_{e}.t}$$

where *k*_2_ (g/(mg∙h) is the rate constant. The pseudo-second-order model assumes that the chemisorption is a rate-limiting step.

The sigmoidal slogistic kinetic model uses three parameters to describe the S-shaped kinetic curves, which consist of an initial lag phase, followed by a phase with a high adsorption rate, and finally a stationary phase. These parameters are:

(10) $$\;sigmoidal\;model\;\left(Slogistic1\right)\;\;q_{t}=q_{e}\frac{1}{1+exp(-k\left(t-\tau \right))}$$

where *τ* (h) describes the inflection point of the function and corresponds to the lag phase; *k* is the kinetic parameter related to the rate constant of the process; and *q*_e_ (mg/g) is the adsorbed amount at equilibrium, which represents the maximum adsorption capacity of Cr(III) or Mn(II) on the surface of the PVA/PEO/SiO_2_/AuNP nanocomposite membrane.

The sigmoidal dose response kinetic model uses four parameters to describe the S-shaped kinetic curves:

(11) $${sigmoidal\;model\;(Doseresponse)\;q}_{t}=q_{\min}+\frac{q_{e}-q_{\min}}{1+{\;10}^{\left((logEC50-t)*p\right)}}$$

where *q*_min_ (mg/g) and *q*_e_ (mg/g) represent the minimum and maximum (equilibrium) adsorbed amounts of Cr(III) or Mn(II) on the surface of the PVA-PEO-TEOS-AuNP nanocomposite membrane, respectively; log*EC*50 is the time at which 50% of the adsorption capacity is reached; and *p* (a dimensionless parameter) describes the slope of the steep part of the curve and can be related to the rate constant of the process.

The kinetic parameters of the analyzed models, estimated through regression analysis, are summarized in Table 2 while the fitted curves are presented in Figures S8 and S9. To determine the most suitable model, both the coefficient of determination (*R*^2^) and the equilibrium adsorption capacity predicted by the model (*q*_e,calc_) should be taken into account [53]. The pseudo-first-order and pseudo-second-order kinetic models failed to converge when describing the adsorption kinetics of the investigated sorbates. This outcome is unsurprising, given the *S*-shaped behavior of the two experimental kinetic curves shown in Figure 7.

The sigmoidal Slogistic1 model accurately represents the experimental adsorption data for both Cr(III) (*q*_e,exp_ = 0.04169 mg/g; *q*_e,calc_ = 0.04446 mg/g; *R*^2^ = 0.9905) and Mn(II) (*q*_e,exp_ = 0.03981 mg/g; *q*_e,calc_ = 0.04102 mg/g; *R*^2^ = 0.9811). This is clearly illustrated in Figure S8, where the black and red curves correspond to the values predicted by the Slogistic1 model for the sorption of Cr(III) and Mn(II), respectively, onto the hydrogel nanocomposite membrane. Similar sigmoidal kinetic behaviors have been observed in the chemisorption of CO_2_ on metal-organic frameworks [54], as well as in autocatalytic [55] and autoinductive [56] chemical reactions.

The estimated parameter *τ* represents the time corresponding to the inflection point of the sorption kinetic curve. This parameter is indirectly linked to the lag phase, as the sorption rate increases until it reaches the inflection point. The slow initial stage of mass transfer for the chemical forms of Cr(III) and Mn(II) formed at the optimal pH of 9 is likely due to internal diffusion resistance. These particles first diffuse rapidly onto the outer membrane surface before slowly penetrating the internal pore surfaces until equilibrium is achieved.

The dose response model, which follows a sigmoidal curve, has been applied in the literature to describe kinetic data in the fields of biology [57], pharmacology [58], and toxicology [59]. This sigmoidal model also accurately describes the experimental results for the adsorption of both Cr(III) (*q*_e,exp_ = 0.04169 mg/g and *q*_e,calc_ = 0.04313 mg/g; *R*^2^ = 0.9945) and Mn(II) (*q*_e,exp_ = 0.04056 mg/g and *q*_e,calc_ = 0.04102 mg/g; *R*^2^ = 0.9803). This can be clearly seen from both the data in Table 2 and Figure S9, where the black and red curves represent the values obtained from the dose response model for the sorption of Cr(III) and Mn(II) ions, respectively, onto the PVA/PEO/SiO_2_/AuNP nanocomposite membrane. However, to the best of our knowledge, this model has not been used in our field of research. For this reason, the physical significance of the model’s parameters requires further investigation and interpretation.

### 2.5. Analytical Applications

The high extraction efficiency of the PVA/PEO/SiO_2_/AuNP nanocomposite membrane demonstrates its potential for the simultaneous speciation analysis of Cr(III)/Cr(VI) and Mn(II)/Mn(VII) in various water samples. Initially, experiments were conducted to determine the maximum sample volume that allows for the quantitative and selective separation of chromium and manganese species. As shown in Table S1, sorption onto the nanocomposite membrane remains quantitative at a sample volume of 20 mL. However, as the sample volume increases, the sorption efficiency decreases, dropping to 64% for Cr(III) and 57% for Mn(II) at a volume of 50 mL. To evaluate the membrane’s applicability for Cr(III)/Cr(VI) determination in surface waters (river, lake, and seawater), they were spiked with a low concentration of Cr(VI) and analyzed using the developed method. The results showed recoveries of Cr(VI) ranging from 95% to 98%, confirming the membrane’s effectiveness in monitoring toxic Cr(VI) levels in surface waters. A key advantage of the synthesized membrane is its ability to simultaneously speciate Cr(III)/Cr(VI) and Mn(II)/Mn(VII) in both treated tap water and disinfected wastewater. Drinking water samples from the municipal water supply in Sofia and wastewater samples from the Kubratovo urban treatment plant were spiked with different concentrations of Cr(VI) and Mn(VII) and analyzed using the described procedure (Section 4.6). The achieved recoveries exceeded 95%, demonstrating the method’s suitability for simultaneous Cr and Mn speciation. Furthermore, the accuracy of the developed analytical procedure was validated using the spike recovery (added/found) method. Water samples were spiked with Cr(III)/Cr(VI) and Mn(II)/Mn(VII) at concentrations relevant to real environmental conditions and analyzed according to the procedure outlined in Section 4.6. The results, presented in Table 3, confirm that the method provides accurate and reliable measurements, reinforcing its applicability for environmental monitoring.

As far as certified reference materials for the content of Cr(III)/Cr(VI) and Mn(II)/Mn(VII) in waters are not available, the developed procedure was applied to the total content of Cr and Mn in SLRS-6: River Water Certified Reference Material for Trace Metals and other Constituents. The results shown in Table 4 are in very good agreement with certified values, additionally supporting the accuracy of the developed procedure.

### 2.6. Analytical Figures of Merit

Analytical figures of merit were defined after the analysis of five parallel samples of tap water from the drinking water treatment plant Bistritsa spiked with 0.5 µg/L Cr(VI) and 0.2 µg/L Mn(VII) and wastewater from the urban wastewater treatment plant Kubratovo spiked with 50 µg/L Cr(VI) and 100 µg/L Mn(VII), according to the developed analytical procedure (Paragraph 4.7) (see Table 5). The relative standard deviation of the results for Cr(III) and Mn(II) varied in the range 3–9% and for Cr(VI) and Mn(VII) in the range 4–10%. The detection limit and quantification limits were calculated based on 3σ and 10σ criteria. The detection and determination limits achieved, as well as relative standard deviations, are depicted in Table 5.

The repeatability of the membrane synthesis procedure was examined by the parallel analysis of spiked tap water samples with membranes prepared by different batches following the developed procedure. The results obtained for the RSD values for all studied chemical species (Cr(III)/Cr(VI) and Mn(II)/Mn(VII)) are identical with these presented in Table 4, indicating very good membrane performance, most probably because for each experiment a newly synthesized membrane is used.

The developed analytical procedure was applied in the national monitoring according to the requirements of the state regulation for the determination of the specific pollutant Cr(VI) in surface waters with a total chromium content above 5 µg/L. The results obtained showed that the annual average concentrations for Cr (VI) are below the accepted national EQS (environmental quality standard). The content of Mn(VII) was determined in tap waters after disinfection (Sofia, Plovdiv, domestic well waters) and it was confirmed that, in all cases, the levels of Mn(VII) were below the determination limit.

A comparison of analytical figures of merit reported in the literature for Cr and Mn speciation procedures using different sorbent materials is presented in Table S2 [22,60,61,62,63,64,65,66,67,68,69,70,71,72,73]. As can be seen, the proposed analytical method in this work for the selective determination of Cr(III) and Cr(VI), as well as Mn(II) and Mn(VII), ensures the comparable detection limits and allows determination of environmentally relevant concentrations of Cr and Mn species in drinking water and wastewater samples.

## 3. Conclusions

A novel PVA/PEO/SiO_2_/AuNP nanocomposite membrane was synthesized, characterized, and proposed as an efficient nanosorbent for the simultaneous speciation of Cr(III)/Cr(VI) and Mn(II)/Mn(VII) in tap water and wastewater. A simple analytical method was developed for the direct determination of toxic Cr(VI) and Mn(VII) following the selective sorption of Cr(III) and Mn(II) onto the membrane. The procedure is highly straightforward, eliminating the need for filtration, centrifugation, or oxidation-reduction steps typically required in the speciation analysis of these elements. The achieved detection and determination limits make this method suitable for routine analytical applications and highly effective for environmental monitoring studies.

## 4. Materials and Methods

### 4.1. Materials, Reagents, and Instruments

All reagents were of an analytical-reagent grade and all aqueous solutions were prepared in high-purity water (Millipore Corp., Milford, MA, USA). Stock standard solutions for Cr and Mn (TraceCERT^®^, 1 g/L in nitric acid (nominal concentration), Merck KGaA, Darmstadt, Germany) were used for the preparation of working diluted standard solutions in 0.5 mol L^−1^ HNO_3_. The stock standard solutions for Mn(VII) and Cr(VI) were prepared by dissolving appropriate amounts of KMnO_4_ and K_2_Cr_2_O_7_ (Merck, Darmstadt, Germany), respectively, in high-purity water. The concentrations of these solutions were further confirmed by flame AAS. CRM: SLRS-6: River Water Certified Reference Material for Trace Metals and other Constituents.

Tetraethylorthosilicate (TEOS, 99%, Fluka, Neu-Ulm, Germany), hydrochloric acid (37%, Merck, Darmstadt, Germany), absolute ethanol (EtOH, 99.8%, Sigma-Aldrich, St. Louis, MO, USA), poly(vinyl alcohol) (PVA, 72,000) and poly(ethylene oxide) (PEO, 400) (Merck, Darmstadt, Germany), and doubly distilled water were used to prepare the PVA/PEO/SiO_2_ hybrid polymer matrix.

Tetrachloroauric (III) acid (HAuCl_4_.3H_2_O, 99%, Panreac Química S.A., Barcelona, Spain), soluble starch (Merck, Darmstadt, Germany), pharmaceutical grade D-(+) glucose, and sodium hydroxide (NaOH, 99%, Merck, Darmstadt, Germany) were used to prepare the aqueous dispersion of starch-coated gold nanoparticles.

Electrothermal AAS measurements of the Cr and Mn were performed with a Perkin Elmer AAnalyst 400 atomic absorption spectrometer (Perkin Elmer, Waltham, MA, USA) on a HGA 900 graphite furnace using an AS 900 autosampler. The sample aliquots of 50 μL were injected into pyrolytic graphite tubes with integrated platforms. The temperature program used consists of a drying step at 120 °C, a pretreatment step at 1000 °C (Mn) and 1300 °C (Cr), and an atomization step at 2300 °C (Mn) and 2600 °C (Cr). Integrated absorbance signals (three replicates) were used for Cr and Mn quantification against external calibration.

UV-Vis absorption spectra of aqueous dispersions of nanoparticles were recorded on an Evolution 300 spectrometer (Thermo Scientific, Waltham, MA, USA) in the range 190–1100 nm, using quartz cuvettes with a 1 cm optical path length. Double-distilled water was used as a reference sample for background absorption. The morphology and particle sizes were examined using a transmission electron microscope (TEM, JEOL JEM-2100 operating at 200 kV) and a scanning electron microscope (SEM, JEOL JSM-5510 operating at 10 kV). X-ray diffraction (XRD) measurement was carried out on an X-ray powder diffractometer, Siemens D500, equipped with the CuKα radiation (λ = 1.54 Å) in 2θ ranging from 15° to 90°. Zeta (ζ) potentials were measured with a Zetasizer Nano ZS (Malvern, PA, USA) instrument.

A digital ultrasonic bath (100 W, 38 kHz, Model DU-32) was used for the ultrasound-assisted preparation of noble metal nanoparticles. The multi-speed vortex MSV-3500 (Biosan, Lishui, China) was used for ensuring vigorous shaking during the sorption of Cr(III) and Mn(II) onto hybrid nanocomposite membranes. The Hettich EBA 20 centrifuge was used for the isolation of membrane sorbents from sample volumes. A microprocessor pH meter (Hanna Instruments, Amorim, Portugal) was used for pH measurements.

### 4.2. Synthesis of Starch-Coated AuNPs

The aqueous dispersions of gold nanoparticles were prepared by a completely green synthesis method based on the reduction of AuCl_4_^−^ using D-(+) glucose as an environmentally benign, soft reductant and soluble starch as a non-toxic capping agent [74].

The reduction of HAuCl_4_ was carried out in an ultrasonic bath under basic reaction conditions. The synthesis procedure for AuNPs, schematically presented in Figure S1, is quite similar to that for AgNPs, as described in detail in our previous studies [75,76]. The resulting aqueous dispersions of gold nanoparticles were stored in the dark at room temperature for subsequent experiments. The wine-red dispersion of AuNPs was stable for several months under storage conditions.

### 4.3. Preparation of the PVA/PEO/SiO_2_/AuNP Nanocomposite Membrane

In order to prepare the hybrid polymer matrix (PVA/PEO/SiO_2_), two initial solutions were prepared:

*Solution 1:* 35.0 g 4% PVA aqueous solution and 0.2 g PEO were mixed in a 100 mL beaker. The resulting solution was homogenized for 5 min;

*Solution 2:* 4.2 g 96% C_2_H₅OH and 1.4 g TEOS were mixed in a 100 mL beaker. The resulting solution was homogenized for 1 min, after which 1 mol/L HCl was added dropwise until the pH reached 2–3. The resulting clear solution of silica sol was then homogenized for 30 min.

Solution 2 was slowly added dropwise to Solution 1 under electromagnetic stirring and a precursor solution (Solution 3) for preparing the hybrid polymer membrane PVA/PEO/SiO_2_ was obtained.

To modify the Solution 3 with AuNPs and obtain the PVA/PEO/SiO_2_/AuNP nanocomposite membrane, a 5.0 mL aqueous dispersion of starch-coated AuNPs was added to the 2.5 mL Solution 3 and placed in a 3 cm diameter cup. The resulting precursor solution (Solution 4) for preparing the PVA/PEO/SiO_2_/AuNP nanocomposite membrane was homogenized via electromagnetic stirring for 15 min and the solvent was slowly evaporated at a constant temperature of 50 °C for 15 h until a dry PVA/PEO/SiO_2_/AuNP nanocomposite membrane was obtained, which can easily be removed from the bottom of the cup. Figure S2 schematically represents the procedure for obtaining the PVA/PEO/SiO_2_/AuNP nanocomposite membrane. For comparison, a PVA/PEO/SiO_2_ hybrid гщaиджeв membrane without AuNPs was also prepared in a similar way using the precursor (Solution 3).

### 4.4. Static Adsorption/Desorption Experiments

The model solutions of 10 mL high-purity water containing 1 µg Cr(IIII), Cr(VI), Mn(II), or Mn(VII) were prepared and adjusted to pH values between 3 and 9 with 1 mol/L HCl or conc. NH_3_ solution. The PVA/PEO/SiO_2_/AuNP or PVA/PEO/SiO_2_ hybrid hydrogel membranes were immersed in these solutions and shaken gently with an electric shaker for 12 h at a temperature of 25 ± 1 °C. After sorption, the membrane was carefully removed and the remaining solution (effluate) was analyzed for Cr and Mn content by ETAAS.

The degree of sorption (*D*_S_, %) of Cr(III) or Mn(II) was calculated by the following equation:

(12) $$D_{S}=\frac{A_{i}–A_{eff}}{A_{i}}\times 100$$

where *A*_i_ (µg) is the initial amount of Cr(III) or Mn(II) in contact with the membrane and *A*_eff_ (µg) is the amount of Cr(III) or Mn(II) in the effluate solution after membrane removal.

The membrane was washed twice with high-purity water and eluted with different eluent solutions or completely dissolved with 1 mL aqua regia under gentle heating (~40 °C) for 20 min. The content of Cr and Mn in the eluate was measured by ETAAS:

(13) $$D_{E}=\frac{A_{el}\;}{A_{i}–A_{eff}}\times 100$$

*A*_el_ (µg) is the amount of Cr(III) or Mn(II) in the eluate.

### 4.5. Isotherm and Kinetic Studies

The following procedure was used for determination of the adsorption capacities of the PVA/PEO/SiO_2_/AuNP hybrid hydrogel membrane: 5 mL solutions (pH 9) with various concentrations of Cr(III) ions (from 0.5 to 10 mg/L) were added to one tested membrane and shaken for 12 h at a temperature of 25 °C. The Cr or Mn concentrations were measured in the effluate solutions by ETAAS under optimized instrumental parameters. All the experiments were performed in triplicate and the average value was used to calculate the maximum adsorption capacity of the PVA/PEO/SiO_2_/AuNP hydrogel membrane (*Q*_e,max_) using the following equation:

(14) $$Q_{e,max}=\frac{\left(C_{0}–C_{e}\right)\times V\;}{m}\;$$

where *Q*_e,max_ (mg/g) is the mass of Cr(III) ions adsorbed per unit mass of the membrane; *V* (L) is the solution volume; *m* (g) is the mass of the membrane; and *C*_0_ and *C*_e_ (mg/L) are the initial and equilibrium concentrations of Cr(III) or Mn(II) ions in the solution, respectively.

The sorption kinetics of Cr(III) or Mn(II) was investigated using one PVA/PEO/SiO_2_/AuNP hybrid hydrogel membrane in contact with 5 mL 1 mg/L Cr(III) or Mn(II) standard solution at pH 9 placed in 15 mL centrifuge tubes on an electrical shaker at 150 rpm at a temperature of 25 ± 1 °C. The sorption time was varied in the range of 1–16 h and the residual Cr or Mn content in the effluate solutions was determined by ETAAS. Each experiment was repeated in triplicate. The amount of Cr(III) or Mn(II) adsorbed at time *t* (h), *q*_t_ (mg/g), was calculated from Equation (15) by the difference between the initial chromium concentration in the solution (*C*_i_, mg/L) at *t* = 0 and the residual chromium concentration at *t* adsorption time (*C*_t_, mg/L):

(15) $$q_{t}=\frac{{(C}_{i}‒C_{t})\times V\;}{m}$$

### 4.6. Analytical Procedure

A water sample of about 20 mL (filtered through a 0.45 µm filter according to the sampling standard) is transferred in a glass beaker, the pH is adjusted to 9, and the PVA/PEO/SiO_2_/AuNP hybrid hydrogel membrane (diameter 2 cm) is immersed in the solution. The solution is gently shaken for 12 h and the membrane is carefully removed by plastic tweezers and transferred to a centrifuge tube. The toxic species Cr(VI) and Mn(VII) are measured in the efluate solution after membrane removal. The membrane sorbent is washed twice with distilled water and dissolved with 1 mL aqua regia under mild heating (~40 °C) for 15 min. The solution obtained is made up to 5 mL with distilled water. Electrothermal AAS is used for Cr and Mn quantification in efluate and eluate solutions if only the speciation of Cr and Mn has to be performed. The developed procedure might be incorporated in the monitoring studies for the quality control of tap and wastewaters and, in such cases, ICP-MS is used for the measurement of the total content of chemical elements of interest (Cd, Pb, Ni, Fe, Zn, Cr, Mn, etc.) and only toxic Cr(VI) and Mn(VII) were determined in a parallel sample in the efluate solution. The amount of Cr(III) and Mn(II) is determined by simple subtraction. The proposed procedure for Cr(VI) and Mn(VII) determination might be performed during sampling—the parallel filtered sample is added to a polypropylene vessel with an inserted membrane. Efluate after sorption is analyzed for Cr or Mn later in the laboratory, together with total element content.

## Figures and Tables

**Table 1:** Langmuir, Freundlich, and DKR isotherm parameters obtained by linear fitting of experimental data for the sorption of Cr(III) and Mn(II) ions onto the PVA/PEO/SiO_2_/AuNP nanocomposite membrane.

Isotherm Model	Parameters	Cr(III)	Mn(II)
Langmuir	*Q*_m_, mg/g	0.2149	0.2256
	*K*_L_, L/mg	71.66	154.8
	*R*_L_ (at *C*_0_ = 1 mg/L)	0 < 0.0138 < 1	0 < 0.0064 < 1
	*R* ^2^	0.9998	0.9999
Freundlich	*K*_F_, mg/g	0.1964	0.2126
	1/*n*	0.0908 << 1	0.0632 << 1
	*R* ^2^	0.7748	0.8127
DKR	*X*_m_, mg/g	0.2159	0.2161
	*β*, mol^2^/J^2^	1.156 × 10^−8^	2.192 × 10^−11^
	*E*, J/mol	6.578	151.0
	*R* ^2^	0.9900	0.8858

**Table 2:** Fitted kinetic parameters of the pseudo-first-order, pseudo-second-order, sigmoidal model (slogistic1), and sigmoidal model (dose response) for the adsorption of Cr(III) and Mn(II) ions onto the PVA/PEO/SiO_2_/AuNP nanocomposite membrane at a concentration of 1 mg/L, pH of 9, temperature of 25 °C, and adsorbent dose (one membrane) of 0.1293 g.

Kinetic Sigmoidal Model	Parameters	Cr(III)	Mn(II)
Slogistic1	*q*_e, calc_, mg/g	0.04446	0.04102
	*τ*, h	8.895	8.301
	*k*, dimensionless	0.5115	0.6566
	*R* ^2^	0.9905	0.9811
Dose response	*q*_e, calc_, mg/g	0.04313	0.04056
	*q*_min, calc_, mg/g	0.0025	0.00144
	log*EC_50_*, h	9.087	8.443
	*p*, dimensionless	0.2840	0.3310
	*R* ^2^	0.9945	0.9803

**Table 3:** Results from the added/found method applied to surface waters, tap waters, and wastewaters.

Sample	Cr(III)	Cr(VI)	Mn (II)	Mn(VII)
River Iskar, µg/L	0.15 ± 0.06	<DL	4.54 ± 0.5	n.a.
Added, µg/L		0.010		
Found, µg/L	0.14 ± 0.08	0.009 ± 0.008	4.32 ± 0.06	
Tap water, Bistritsa, µg/L	0.092 ± 0.008	<DL	15.4 ± 0.5	<DL
Added, µg/L		0.05		2.00
Found, µg/L	0.093 ± 0.009	0.052 ± 0.007	14.9 ± 0.5	1.95 ± 0.45
Wastewater	1.23 ± 0.09	0.034 ± 0.004	256 ± 25	21 ± 2
Added, µg/L		0.10		10
Found, µg/L	1.34 ± 0.07	0.14 ± 0.006	249 ± 23	32 ± 3

**Table 4:** Analysis of SLRS-6: River Water Certified Reference Material.

	Cr (Cr(III) + Cr(VI))	Mn (Mn(II) + Mn(VII))
Certified value, µg/L	0.252 ± 0.012	2.12 ± 0.10
Proposed procedure, µg/L	0.245 ± 0.016	2.04 ± 0.09
Recovery, %	97.2 ± 0.3	96.2 ± 0.2

**Table 5:** Analytical figures of merit.

Parameters	Cr(III)	Cr(VI)	Mn(II)	Mn(VII)
Detection limit, µg/L	0.09	0.1	0.04	0.05
Determination limit, µg/L	0.26	0.3	0.12	0.15
RSD, % in tap water for the species content (LOD-100 µg/L)	3–7	4–7	3–7	4–7
RSD, % in wastewater for the species content (LOD-500 µg/L)	4–10	3–9	3–10	3–8

## Data Availability

The original contributions presented in this study are included in the article/Supplementary Material. Further inquiries can be directed to the corresponding authors.

## Supplementary Materials

The following supporting information can be downloaded at: https://www.mdpi.com/article/10.3390/gels11030205/s1, Figure S1: Scheme of the synthesis procedure for obtaining starch-coated gold nanoparticles; Figure S2: A schematic representation of the procedure for preparing hybrid composite hydrogel membranes; Figure S3: X-ray diffraction patterns of starch-coated AuNPs sample; Figure S4: TEM of PVA/PEO/SiO_2_/AuNPs hybrid nanocomposite membrane and the corresponding TEM-EDS elemental images; Figure S5: Comparison of degree of sorption (*D*s, %) of (a) Cr(III) ions and (b) Mn(II) ions using PVA/PEO/SiO_2_ or PVA/PEO/SiO_2_/AuNPs membranes as sorbents at pH 3 and pH 9; temperature 25 °C; adsorbent dose is one membrane with diameter 2.5 cm—0.1293 g; Figure S6: Various isotherm models (Langmuir, Freundlich, DKR (Dubinin–Kagaber–Radushkevich) and Temkin) for adsorption of Cr(III) onto PVA/PEO/SiO_2_/AuNPs nanocomposite membrane at optimum pH 9, temperature 25 °C and adsorbent dose (one membrane with diameter 2.5 cm)—0.1293 g; Figure S7: Various isotherm models (Langmuir, Freundlich, DKR (Dubinin–Kagaber–Radushkevich) and Temkin) for adsorption of Mn(II) onto PVA/PEO/SiO_2_/AuNPs nanocomposite membrane at optimum pH 9, temperature 25 °C and adsorbent dose (one membrane with diameter 2.5 cm)—0.1293 g; Figure S8: Sigmoidal kinetic adsorption model (slogistic1) for adsorption of (a) Cr(III) and (b) Mn(II) onto PVA/PEO/SiO_2_/AuNPs nanocomposite membrane (*c*_0_(Cr(III)) = 1 mg/L (19.23 μmol/L), *c*_0_(Mn(II)) = 1 mg/L (18.20 μmol/L)); pH 9; temperature 25 °C); Figure S9: Sigmoidal kinetic adsorption model (dose response) for adsorption of (a) Cr(III) and (b) Mn(II) onto PVA/PEO/SiO_2_/AuNPs nanocomposite membrane (*c*_0_(Cr(III)) = 1 mg/L (19.23 μmol/L), *c*_0_(Mn(II)) = 1 mg/L (18.20 μmol/L)); pH 9; temperature 25 °C); Table S1. Effect of sample volume on the sorption degree of Cr(III) and Mn(II) onto the PVA/PEO/SiO_2_TEOS/AuNPs membrane sorbent; pH 9, sorption time of 16 h, adsorbent dose (one membrane with diameter 2.5 cm)—0.1293 g; Table S2. Comparison of SPE procedure for simultaneous speciation of Cr(III)/Cr(VI) and Mn(II)/Mn(VII) utilizing PVA/PEO/SiO_2_/AuNPs nanocomposite membrane with other reported methods for speciation of Cr(III)/Cr(VI) or Mn(II)/Mn(VII).

## References

[1] Metze D., Jakubowski N., Klockow D., Cornelis R., Crews H., Caruso J., Heumann K.G. (2005). Species in the environment, food, medicine & occupational health. Handbook of Elemental Speciation II: Species in the Environment, Food, Medicine and Occupational Health.

[2] Kot A., Namiesńik J. (2000). The role of speciation in analytical chemistry. TrAC Trends Anal. Chem..

[3] Llaver M., Fiorentini E.F., Oviedo M.N., Quintas P.Y., Wuilloud R.G. (2021). Elemental Speciation Analysis in Environmental Studies: Latest Trends and Ecological Impact. Int. J. Environ. Res. Public Health.

[4] Rai D., Eary L.E., Zachara J.M. (1989). Environmental chemistry of chromium. Sci. Total Environ..

[5] Richard F.C., Bourg A.C. (1991). Aqueous geochemistry of chromium: A review. Water Res..

[6] Kotaś J., Stasicka Z. (2000). Chromium occurrence in the environment and methods of its speciation. Environ. Pollut..

[7] Chebeir M., Chen G., Liu H. (2016). Emerging investigators series: Frontier review: Occurrence and speciation of chromium in drinking water distribution systems. Environ. Sci.:Water Res. Technol..

[8] Katz S.A., Salem H. (1993). The toxicology of chromium with respect to its chemical speciation—A review. J. Appl. Toxicol..

[9] Saha R., Nandi R., Saha B. (2011). Sources and toxicity of hexavalent chromium. J. Coord. Chem..

[10] Jablońska-Czapla M. (2015). Manganese and its speciation in environmental samples using hyphenated techniques: A review. J. Elem..

[11] Witholt R., Gwiazda R.H., Smith D.R. (2000). The neurobehavioral effects of sub-chronic manganese exposure in the presence and absence of pre-parkinsonism. Neurotoxicol. Teratol..

[12] Yokel R.A., Crossgrove J.S., Bukaveckas B.L. (2003). Manganese distribution across the blood-brain barrier. II. Manganese efflux from the brain does not appear to be carrier mediated. NeuroToxicology.

[13] Gómez V., Callao M.P. (2006). Chromium determination and speciation since 2000. TrAC Trends Anal. Chem..

[14] Namieśnik J., Rabajczyk A. (2011). Speciation Analysis of Chromium in Environmental Samples. Crit. Rev. Environ. Sci. Technol..

[15] Rakhunde R., Deshpande L., Juneja H.D. (2012). Chemical Speciation of Chromium in Water: A Review. Crit. Rev. Environ. Sci. Technol..

[16] Dawra N., Dabas N. (2022). Advances in spectrophotometric determination of Chromium (III) and Chromium(VI) in water: A review. J. Environ. Anal. Chem..

[17] Markiewicz B., Komorowicz I., Sajnóg A., Belter M., Barałkiewicz D. (2015). Chromium and its speciation in water samples by HPLC/ICP-MS—Technique establishing metrological traceability: A review since 2000. Talanta.

[18] Rumsby P., Rockett L., Clegg H., Jonsson J., Benson V., Harman M., Doyle T., Rushton L., Wilkinson D., Warwick P. (2014). Speciation of manganese in drinking water. Toxicol. Lett..

[19] Pearson G.F., Greenway G.M. (2005). Recent developments in manganese speciation. TrAC Trends Analyt Chem..

[20] Grygo-Szymanko E., Tobiasz A., Walas S. (2016). Speciation analysis and fractionation of manganese—A review. TrAC Trends Anal. Chem..

[21] Zhang M., Zhan G., Chen Z. (2005). Iodometric Amplification Method for the Determinations of Microgram Amounts of Manganese(II), Manganese(VII), Chromium(III) and Chromium(VI) in Aqueous Solution. Anal. Sci..

[22] Abdolmohammad-Zadeh H., Sadeghi G.H. (2012). A nano-structured material for reliable speciation of chromium and manganese in drinking waters, surface waters and industrial wastewater effluents. Talanta.

[23] Kolekar A.G., Nille O.S., Gunjal D.B., Naik V.M., Ngoc Q.N., Sohn D., Kolekar G.B., Gokavi G.S., More V.R. (2024). Prompt in situ synthesis of sulphur doped carbon dots from jaggery for parallel determination of iron, chromium and manganese in environmental samples. J. Photochem. Photobiol. A Chem..

[24] Wakshe S.B., Dongare P.R., Gore A.H., Mote G.V., Salunkhe S.Y., Mahanwar S.T., Anbhule P.V., Kolekar G.B. (2021). A highly sensitive and selective phthalazine derivative based fluorescent organic nanosheets for simultaneous detection of Cr^6+^ and Mn^7+^ in aqueous media. Inorg. Chim. Acta.

[25] Hagarová I., Nemček L. (2021). Application of Metallic Nanoparticles and Their Hybrids as Innovative Sorbents for Separation and Pre-concentration of Trace Elements by Dispersive Micro-Solid Phase Extraction: A Mini review. Front. Chem..

[26] Hua M., Zhang S., Pan B., Zhang W., Lv L., Zhang Q. (2012). Heavy metal removal from water/wastewater by nanosized metal oxides: A review. J. Hazard. Mater..

[27] Li Y.K., Wang X.Y., Liu X., Yang T., Chen M.L., Wang J.H. (2020). Ensuring high selectivity for preconcentration and detection of ultra-trace cadmium using a phage-functionalized metal–organic framework. Analyst.

[28] Zhang Y., Wu B., Xu H., Liu H., Wang M., He Y., Pan B. (2016). Nanomaterials-enabled water and wastewater treatment. NanoImpact.

[29] Herrero-Latorre C., Barciela-García J., García-Martín S., Pena-Crecente R.M. (2018). Graphene and carbon nanotubes as solid phase extraction sorbents for the speciation of chromium: A review. Anal. Chim. Acta.

[30] Hasanpour M., Hatami M. (2020). Application of three dimensional porous aerogels as adsorbent for removal of heavy metal ions from water/wastewater: A review study. Adv. Colloid Interface Sci..

[31] Sajid M., Basheer C. (2016). Layered double hydroxides: Emerging sorbent materials for analytical extractions. TrAC Trends Anal. Chem..

[32] Li Y.K., Yang T., Chen M.L., Wang J.H. (2021). Recent advances in nanomaterials for analysis of trace heavy metals. Crit. Rev. Anal. Chem..

[33] He M., Huang L., Zhao B., Chen B., Hu B. (2017). Advanced functional materials in solid phase extraction for ICP-MS determination of trace elements and their species-A review. Anal. Chim. Acta.

[34] Hemmati M., Rajabi M., Asghari A. (2018). Magnetic nanoparticle based solid-phase extraction of heavy metal ions: A review on recent advances. Microchim. Acta.

[35] Samiey B., Cheng C.H., Wu J. (2014). Organic-inorganic hybrid polymers as adsorbents for removal of heavy metal ions from solutions: A review. Materials.

[36] Rivas B.L., Urbano B.F., Sánchez J. (2018). Water-soluble and insoluble polymers, nanoparticles, nanocomposites and hybrids with ability to remove hazardous inorganic pollutants in water. Front. Chem..

[37] Shamsipur M., Fasihi J., Ashtari K. (2007). Grafting of ion-imprinted polymers on the surface of silica gel particles through covalently surface-bound initiators: A selective sorbent for uranyl ion. Anal. Chem..

[38] Buhani B., Narsito N., Nuryono N., Kunarti E.S. (2010). Production of metal ion imprinted polymer from mercapto-silica through sol–gel process as selective adsorbent of cadmium. Desalination.

[39] Li F., Jiang H., Zhang S. (2007). An ion-imprinted silica-supported organic–inorganic hybrid sorbent prepared by a surface imprinting technique combined with a polysaccharide incorporated sol–gel process for selective separation of cadmium(II) from aqueous solution. Talanta.

[40] Djerahov L., Vasileva P., Karadjova I. (2016). Self-standing chitosan film loaded with silver nanoparticles as a tool for selective determination of Cr (VI) by ICP-MS. Microchem. J..

[41] Vimala K., Murali Mohana Y., Samba Sivudu K., Varaprasad K., Ravindra S., Narayana Reddy N., Padma Y., Sreedhar B., Mohana Raju K. (2010). Fabrication of porous chitosan films impregnated with silver nanoparticles: A facile approach for superior antibacterial application. Colloids Surf. B Biointerfaces.

[42] Ščančar J., Milačič R. (2014). A critical overview of Cr speciation analysis based on high performance liquid chromatography and spectrometric techniques. J. Anal. At. Spectrom..

[43] Mladenova E.K., Dakova I.G., Karadjova I.B. (2011). Chitosan membranes as sorbents for trace elements determination in surface waters. Environ Sci. Pollut. Res..

[44] Chen X., Hossain M.F., Duan C., Lu J., Tsang Y.F., Islam M.S., Zhou Y. (2022). Isotherm models for adsorption of heavy metals from water—A review. Chemosphere.

[45] Al-Ghouti M.A., Da’ana D.A. (2020). Guidelines for the use and interpretation of adsorption isotherm models: A review. J. Hazard. Mater..

[46] Priastomo Y., Setiawan H.R., Kurniawan Y.S., Ohto K. (2020). Simultaneous removal of lead (II), chromium (III), and copper (II) heavy metal ions through an adsorption process using C-phenylcalix [4] pyrogallolarene material. J. Environ. Chem. Eng..

[47] Abdelwahab O., Fouad Y.O., Amin N.K., Mandor H. (2015). Kinetic and thermodynamic aspects of cadmium adsorption onto raw and activated guava (*Psidium guajava*) leaves. Environ. Prog. Sustain..

[48] Samadi N., Hasanzadeh R., Rasad M. (2015). Adsorption isotherms, kinetic, and desorption studies on removal of toxic metal ions from aqueous solutions by polymeric adsorbent. J. Appl. Polym. Sci..

[49] Embaby M.A., Moniem S.M., Fathy N.A., El-Kady A.A. (2021). Nanocarbon hybrid for simultaneous removal of arsenic, iron and manganese ions from aqueous solutions. Heliyon.

[50] Gao X., Guo C., Hao J., Zhao Z., Long H., Li M. (2020). Adsorption of heavy metal ions by sodium alginate based adsorbent—A review and new perspectives. Int. J. Biol. Macromol..

[51] Kaptso K.G., Njintang Y.N., Komnek A.E., Hounhouigan J., Scher J., Mbofung C.M. (2008). Physical properties and rehydration kinetics of two varieties of cowpea (*Vigna unguiculata*) and bambara groundnuts (*Voandzeia subterranea*) seeds. J. Food Eng..

[52] Cai Q., Turner B.D., Sheng D., Sloan S. (2015). The kinetics of fluoride sorption by zeolite: Effects of cadmium, barium and manganese. J. Contam. Hydrol..

[53] Salehi E., Madaeni S.S., Vatanpour V. (2012). Thermodynamic investigation and mathematical modeling of ion-imprinted membrane adsorption. J. Membr. Sci..

[54] Martell J.D., Milner P.J., Siegelman R.L., Long J.R. (2020). Kinetics of cooperative CO_2_ adsorption in diamine-appended variants of the metal–organic framework Mg_2_(dobpdc). Chem. Sci..

[55] Quaranta M., Gehring T., Odell B., Brown J.M., Blackmond D.G. (2010). Unusual inverse temperature dependence on reaction rate in the asymmetric autocatalytic alkylation of pyrimidyl aldehydes. J. Am. Chem. Soc..

[56] Mathew S.P., Klussmann M., Iwamura H., Wells D.H., Armstrong A., Blackmond D.G. (2006). A mechanistic rationalization of unusual kinetic behavior in proline-mediated C–O and C–N bond-forming reactions. Chem. Commun..

[57] Calabrese E., Hacker M., Messer W.S., Bachmann K.A. (2009). Hormesis and pharmacology. Pharmacology: Principles and Practice.

[58] Huang Y.Y., Chen A.C., Carroll J.D., Hamblin M.R. (2009). Biphasic dose response in low level light therapy. Dose-Response.

[59] Newberry N.R., Gilbert M.J. (1989). Biphasic dose-response curve to muscarine on the rat superior cervical ganglion. Eur. J. Pharmacol..

[60] Wu P., Chen H., Cheng G., Hou X. (2009). Exploring surface chemistry of nano-TiO_2_ for automated speciation analysis of Cr(III) and Cr(VI) in drinking water using flow injection and ET-AAS detection. J. Anal. At. Spectrom..

[61] Huang Y.-F., Li Y., Jiang Y., Yan X.-P. (2010). Magnetic immobilization of amine-functionalized magnetite microspheres in a knotted reactor for on-line solid-phase extraction coupled with ICP-MS for speciation analysis of trace chromium. J. Anal. At. Spectrom..

[62] Jiang H., Yang T., Wang Y., Lian H., Hu X. (2013). Magnetic solid-phase extraction combined with graphite furnace atomic absorption spectrometry for speciation of Cr(III) and Cr(VI) in environmental waters. Talanta.

[63] Diniz K.M., Tarley C.R.T. (2015). Speciation analysis of chromium in water samples through sequential combination of dispersive magnetic solid phase extraction using mesoporous amino-functionalized Fe_3_O_4_/SiO_2_ nanoparticles and cloud point extraction. Microchem. J..

[64] Islam A., Ahmad H., Zaidi N., Kumar S. (2015). A graphene oxide decorated with triethylenetetramine-modified magnetite for separation of chromium species prior to their sequential speciation and determination via FAAS. Microchim. Acta..

[65] Heena G., Rani S., Malik A.K., Kabir A., Furton K.G. (2016). Speciation of Cr (III) and Cr (VI) Ions via Fabric Phase Sorptive Extraction for Their Quantification via HPLC with UV Detection. J. Chromatogr. Sep. Tech..

[66] Dakova I., Vasileva P., Karadjova I. (2022). Cr(III) Ion-Imprinted Hydrogel Membrane for Chromium Speciation Analysis in Water Samples. Gels.

[67] Chen S., Zhu L., Lu D., Cheng X., Zhou X. (2010). Separation and chromium speciation by single-wall carbon nanotubes microcolumn and inductively coupled plasma mass spectrometry. Microchim. Acta.

[68] Şahan S., Saçmacı Ş., Kartal Ş., Saçmacı M., Şahin U., Ülgen A. (2014). Development of a new on-line system for the sequential speciation and determination of chromium species in various samples using a combination of chelating and ion exchange resins. Talanta.

[69] Pramanik S., Dey S., Chattopadhyay P. (2007). A new chelating resin containing azophenolcarboxylate functionality: Synthesis, characterization and application to chromium speciation in wastewater. Anal. Chim. Acta.

[70] Chen S., Qin X., Gu W., Zhu X. (2016). Speciation analysis of Mn (II)/Mn (VII) using Fe_3_O_4_@ionic liquids-β-cyclodextrin polymer magnetic solid phase extraction coupled with ICP-OES. Talanta.

[71] Shirkhanloo H., Khaligh A., Zavvar Mousavi H., Rashidi AM. (2016). Ultrasound assisted-dispersive-micro-solid phase extraction based on bulky amino bimodal mesoporous silica nanoparticles for speciation of trace manganese (II)/(VII) ions in water samples. Microchem. J..

[72] Qian A.X.S., He G.H.F., Han X. (2001). Separation and preconcentration of MnVII/MnII speciation on crosslinked chitosan and determination by flame atomic absorption spectrometry. Analyst.

[73] Rakhtshah J., Shirkhanloo H., Mobarake M.D. (2022). Simultaneously speciation and determination of manganese(II) and (VII) ions in water, food, and vegetable samples based on immobilization of N-acetylcysteine on multi-walled carbon nanotubes. Food Chem..

[74] Katti K.K., Kattumuri V., Bhaskaran S., Katti K.V., Kannan R. (2009). Facile and general method for synthesis of sugar-coated gold nanoparticles. Int. J. Green Nanotechnol. Biomed..

[75] Vasileva P., Donkova B., Karadjova I., Dushkin C. (2011). Synthesis of starch-stabilized silver nanoparticles and their application as a surface plasmon resonance-based sensor of hydrogen peroxide. Colloids Surf. A Physicochem. Eng. Asp..

[76] Yordanova T., Vasileva P., Karadjova I. (2020). Noble metal nanocomposites as tools for fast and reliable speciation analysis of mercury in water samples. J. Environ. Anal. Chem..

