# Supplementary material for: Newly Designed Organic-Inorganic Nanocomposite Membrane for Simultaneous Cr and Mn Speciation in Waters
Source: Gels. 2025 Mar 15;11(3):205. doi: 10.3390/gels11030205 (PMC11942063; doi:10.3390/gels11030205)
Supplement: Supplementary file 1 [file gels-11-00205-s001.zip › gels-3518252-supplementary.pdf]

Supplemental material

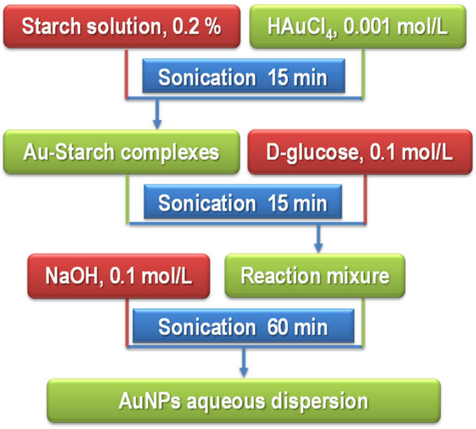

Figure S1. Scheme of the synthesis procedure for obtaining starch-coated gold nanoparticles.

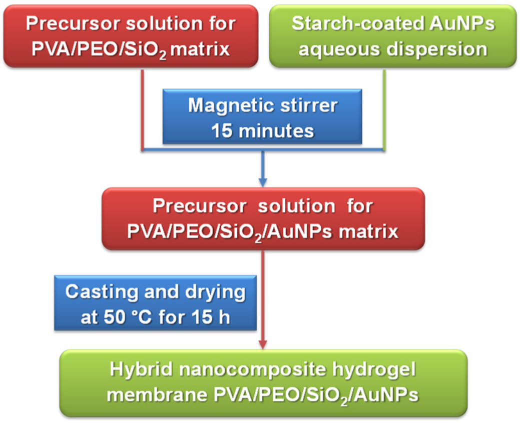

Figure S2. A schematic representation of the procedure for preparing PVA/PEO/SiO_2/AuNPs hybrid nanocomposite hydrogel membrane.

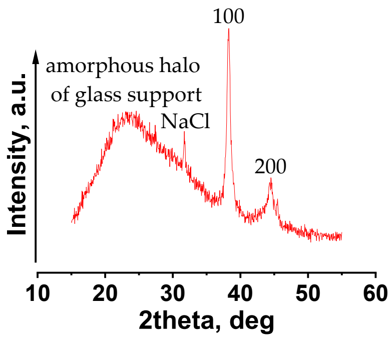

Figure S3. X-ray diffraction patterns of starch-coated AuNPs sample.

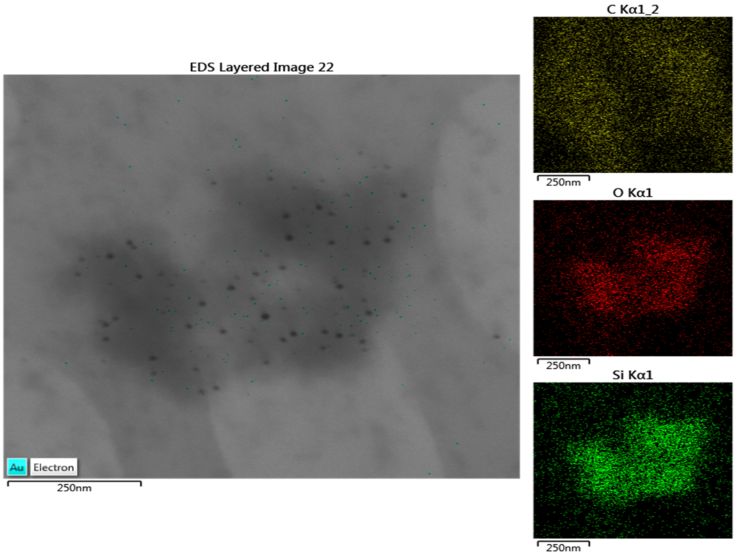

Figure S4. TEM of PVA/PEO/SiO_2/AuNPs hybrid nanocomposite membrane and the corresponding TEM-EDS elemental images

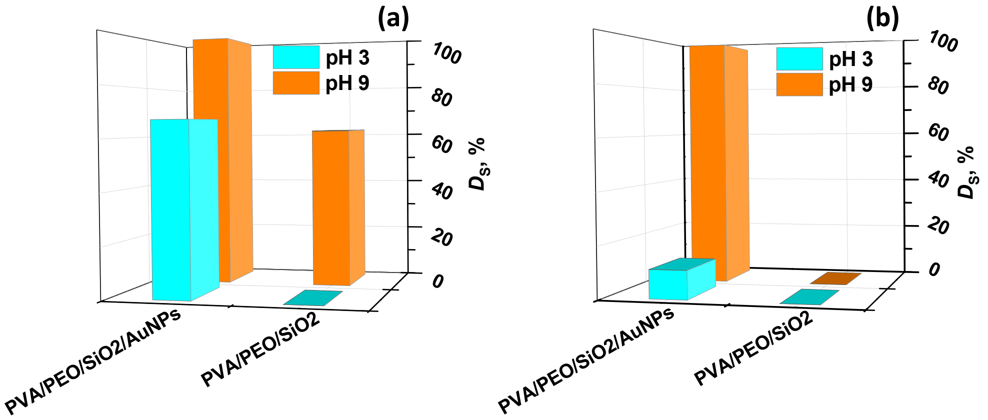

Figure S5. Comparison of degree of sorption (*D*s, %) of (a) Cr(III) ions and (b) Mn(II) ions using PVA/PEO/SiO_2 or PVA/PEO/SiO_2/AuNPs membranes as sorbents at pH 3 and pH 9; temperature 25°C; adsorbent dose is one membrane with diameter 2.5 cm — 0.1293 g.

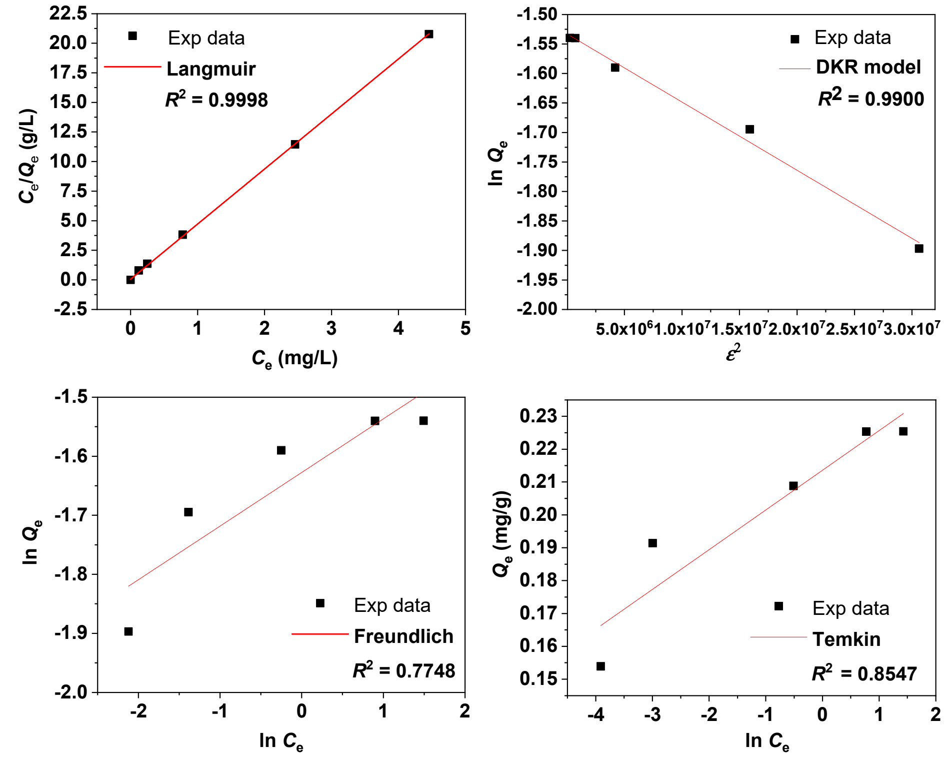

Figure S6. Various isotherm models (Langmuir, Freundlich, DKR (Dubinin-Kagaber-Radushkevich) and Temkin) for adsorption of Cr(III) onto PVA/PEO/SiO_2/AuNPs nanocomposite membrane at optimum pH 9, temperature 25°C and adsorbent dose (one membrane with diameter 2.5 cm) — 0.1293 g.

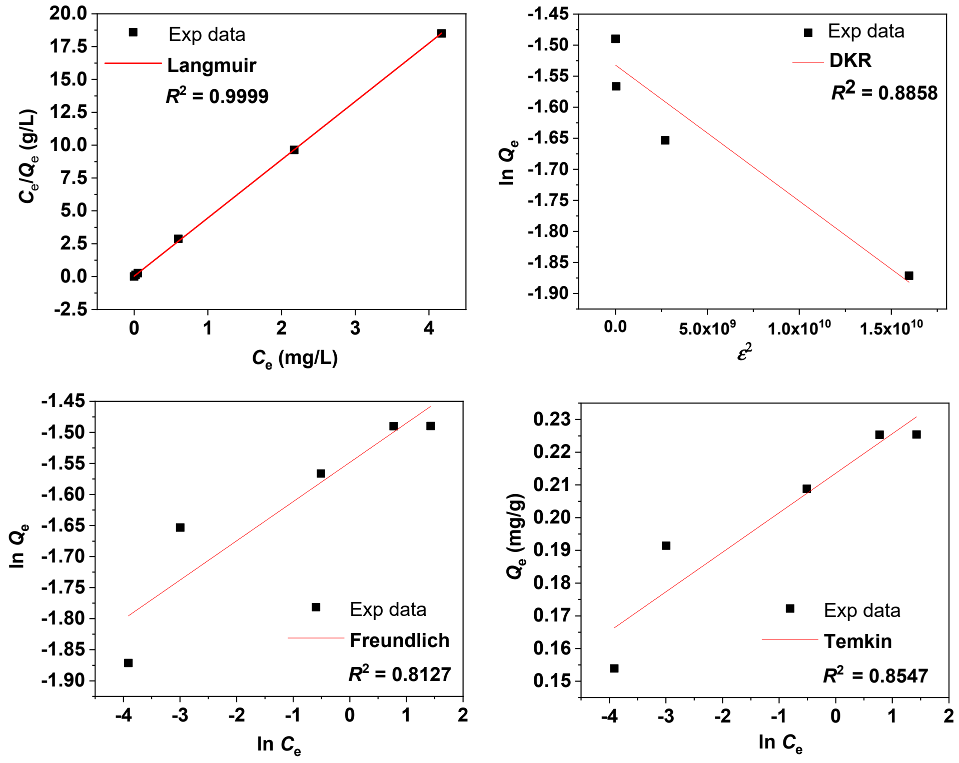

Figure S7. Various isotherm models (Langmuir, Freundlich, DKR (Dubinin-Kagaber-Radushkevich) and Temkin) for adsorption of Mn(II) onto PVA/PEO/SiO_2/AuNPs nanocomposite membrane at optimum pH 9, temperature 25°C and adsorbent dose (one membrane with diameter 2.5 cm) — 0.1293 g.

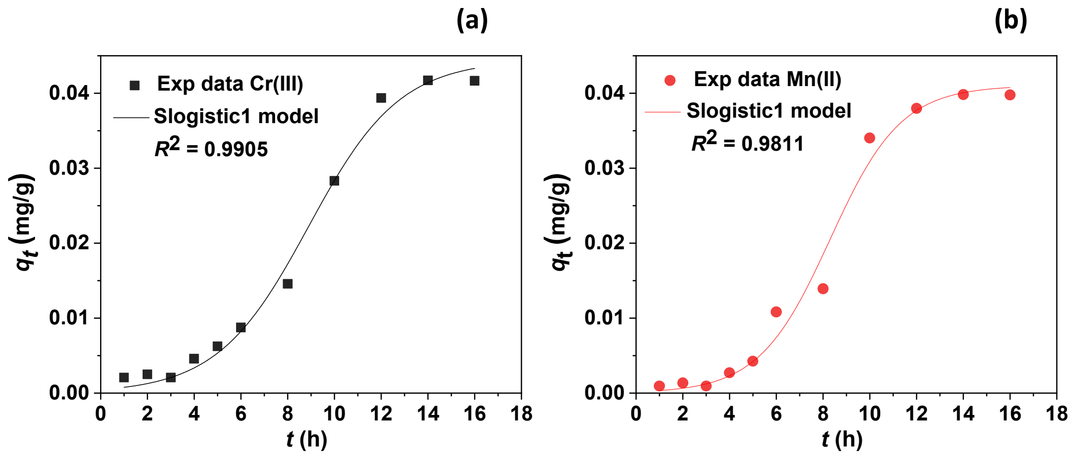

Figure S8. Sigmoidal kinetic adsorption model (slogistic1) for adsorption of (a) Cr(III) and (b) Mn(II) onto PVA/PEO/SiO_2/AuNPs nanocomposite membrane (c_0(Cr(III)) = 1 mg/L (19.23 µmol/L), c_0(Mn(II)) = 1 mg/L (18.20 µmol/L)); pH 9; temperature 25°C).

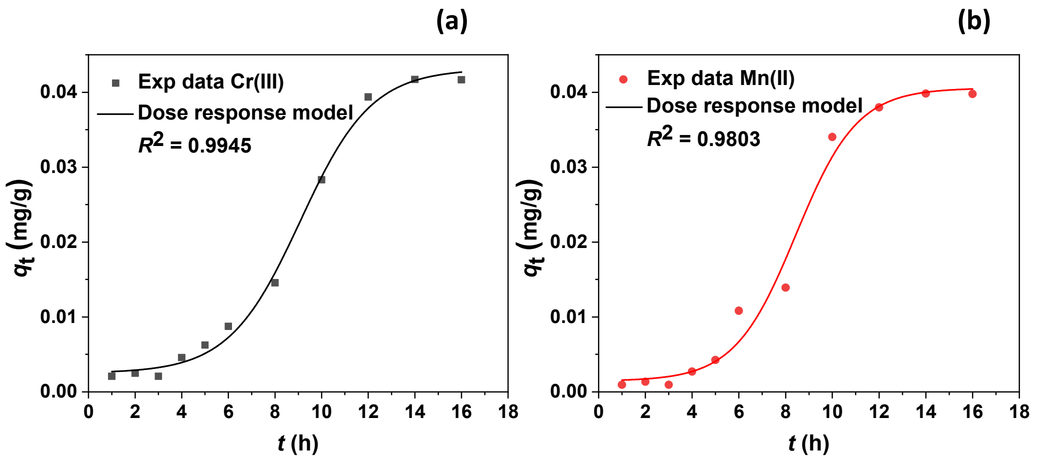

Figure S9. Sigmoidal kinetic adsorption model (dose response) for adsorption of (a) Cr(III) and (b) Mn(II) onto PVA/PEO/SiO_2/AuNPs nanocomposite membrane (c_0(Cr(III)) = 1 mg/L (19.23 µmol/L), c_0(Mn(II)) = 1 mg/L (18.20 µmol/L)); pH 9; temperature 25°C).

Table S1. Effect of sample volume on the sorption degree of Cr(III) and Mn(II) onto the PVA/PEO/SiO_2/AuNPs membrane sorbent; pH 9, sorption time of 16 h, adsorbent dose (one membrane with diameter 2.5 cm) – 0.1293 g.

Sample volume, mL	Ds, % for Cr(III)	Ds, % for Mn(II)
10	97 ± 3[1]	94 ± 2
20	99 ± 2	98 ± 3
30	87 ± 3	82 ± 2
40	78 ± 2	65 ± 3
50	63 ± 4	57 ± 3

[1] Mean ± standard deviation

Table S2. Comparison of SPE procedure for simultaneous speciation of Cr(III)/Cr(VI) and Mn(II)/Mn(VII) utilizing PVA/PEO/SiO_2/AuNPs nanocomposite membrane with other reported methods for speciation of Cr(III)/Cr(VI) or Mn(II)/Mn(VII).

**Adsorbent**	**Species**	**Instrumental method**	**Sample**	**LOD, μg/L**	**Ref.**
Nanometer-sized TiO_2	Cr(VI)<br>Cr(III)	ETAAS	Drinking water	0.01<br>0.006	[60]
Knotted reactor with magnetically immobilized amine-functionalized Fe_3O_4 microspheres	Cr(VI)<br>Cr(III)	ICP-MS	Drinking water	0.0021<br>0.0015	[61]
Zincon-immobilized silica-coated Fe_3O_4	Cr(VI)<br>Cr(III)	ETAAS	Water	0.011<br>0.016	[62]
Mesoporous aminfunctionalized Fe_3O_4 (dispersive MSPE combined with CPE)	Cr(VI)<br>Cr(III)	FAAS	Water<br>Biological samples	1.1<br>3.2	[63]
Graphene oxide, decorated with magnetite modified with triethylenetetramine	Cr(VI)<br>Cr(III)	FAAS	Tannery waste Water, River water, Industry water	1.4<br>1.6	[64]
Cellulose fabric/modified with a sol–gel/polytetrahydrofuran composite	Cr(VI)<br>Cr(III)	HPLC-UV	Ground and drinking water, wastewater	0.003<br>0.001	[65]
Poly(vinylalcohol)/sodium alginate/AuNPs hydrogel membranes	Cr(VI)<br>Cr(III)	ETAAS	Water	0.01<br>0.001	[66]
SWCNTs, oxidized	Cr(VI)<br>Cr(III)	ICP-MS	Natural water<br>Waste waters	0.024<br>0.01	[67]
Poly 2-(5-methylisoxazol) methacrylamide-co-2-acrylamido-2-methyl-1-propanesulfonic acidcodivinyl-benzene and Dowex 21K resin	Cr(VI)<br>Cr(III)	FAAS	Tap water<br>Seawater<br>Spring water<br>Industrial water	0.3<br>0.05	[68]
Polystyrene divinylbenzene copolymer as a chelating resin	Cr(VI)<br>Cr(III)	ETAAS	Wastewater	0.9<br>0.6	[69]
PVA/PEO/SiO_2TEOS/AuNPs hydrogel membrane	Cr(VI)<br>Cr(III)	ETAAS	Tap and waste-water	0.1<br>0.09	This work
β-cyclodextrin/modified with ionic liquid/attached on Fe_3O_4 nanoparticles	Mn(VII)<br>Mn(II)	ICP-OES	Tap, mineral, and lake water	0.27<br>0.15	[70]
Amino bimodal mesoporous silica nanoparticles	Mn(VII)<br>Mn(II)	AT-FAAS	Natural water	0.008<br>0.007	[71]
Crosslinked chitosan	Mn(VII)<br>Mn(II)	FAAS	Environmental water	1.98<br>-	[72]
N-acetylcysteine on multi-walled carbon nanotubes	Mn(VII)<br>Mn(II)	AT-FAAS	Surface water	0.14<br>0.12	[73]
Nickel–aluminum layered double Hydroxide	Cr(VI)<br>Mn(VII)	FAAS	Drinking water, surface water and industrial wastewater	0.51<br>0.47	[22]

PVA/PEO/SiO_2/AuNPs nanocomposite membrane	Mn(VII)<br>Mn(II)	ETAAS	Tap and waste-water	0.05<br>0.04	This work

References

60. Wu, P.; Chen, H.; Cheng, G.; Hou, X. Exploring surface chemistry of nano-TiO_2 for automated speciation analysis of Cr(III) and Cr(VI) in drinking water using flow injection and ET-AAS detection. *J. Anal. At. Spectrom.* **2009**, *24*, 1098.

61. Huang, Y.-F.; Li,Y.; Jiang, Y.; Yan, X.-P. Magnetic immobilization of amine-functionalized magnetite microspheres in a knotted reactor for on-line solid-phase extraction coupled with ICP-MS for speciation analysis of trace chromium. *J. Anal. At. Spectrom.* **2010**, *25*, 1467.

62. Jiang, H.; Yang, T.; Wang, Y. Lian, H.; Hu, X. Magnetic solid-phase extraction combined with graphite furnace atomic absorption spectrometry for speciation of Cr(III) and Cr(VI) in environmental waters. *Talanta* **2013,** *116*, 361–367.

63. Diniz, K.M.; Tarley, C.R.T. Speciation analysis of chromium in water samples through sequential combination of dispersive magnetic solid phase extraction using mesoporous amino-functionalized Fe_3O_4/SiO_2 nanoparticles and cloud point extraction. *Microchem. J.* **2015**, *123*, 185–195.

64. Islam, A.; Ahmad, H.; Zaidi, N.; Kumar, S. A graphene oxide decorated with triethylenetetramine-modified magnetite for separation of chromium species prior to their sequential speciation and determination via FAAS. *Microchim. Acta.* **2015**, *183*, 289–296.

65. Heena, G.; Rani, S.; Malik, A.K.; Kabir, A.; Furton, K.G. Speciation of Cr (III) and Cr (VI) Ions via Fabric Phase Sorptive Extraction for Their Quantification via HPLC with UV Detection. *J. Chromatogr. Sep. Tech.* **2016**, 7, 1–6.

66. Dakova, I.; Vasileva, P.; Karadjova, I. Cr(III) Ion-Imprinted Hydrogel Membrane for Chromium Speciation Analysis in Water Samples. *Gels* **2022**, 8, 757.

67. Chen, S.; Zhu, L.; Lu, D.; Cheng, X.; Zhou, X. Separation and chromium speciation by single-wall carbon nanotubes microcolumn and inductively coupled plasma mass spectrometry, *Microchim. Acta.* **2010**, 169, 123–128.

68. Şahan, S.; Saçmacı,Ş. Kartal, Ş.; Saçmacı, M.; Şahin, U. Ülgen, A. Development of a new on-line system for the sequential speciation and determination of chromium species in various samples using a combination of chelating and ion exchange resins. *Talanta* **2014**, 120, 391–397.

69. Pramanik, S.; Dey,S.; Chattopadhyay, P. A new chelating resin containing azophenolcarboxylate functionality: synthesis, characterization and application to chromium speciation in wastewater. *Anal. Chim. Acta.* **2007**, *584*, 469–476.

70. Chen, S.; Qin, X.; Gu, W.; Zhu, X. Speciation analysis of Mn (II)/Mn (VII) using Fe_3O_4@ionic liquids-β-cyclodextrin polymer magnetic solid phase extraction coupled with ICP-OES. *Talanta* **2016,** *161*, 325–332.

71. Shirkhanloo, H.; Khaligh, A.; Zavvar Mousavi, H.; Rashidi, AM. Ultrasound assisted-dispersive-micro-solid phase extraction based on bulky amino bimodal mesoporous silica nanoparticles for speciation of trace manganese (II)/(VII) ions in water samples. *Microchem. J.* **2016**, *124*, 637–645.

72. Qian, A.X.S.; He, G.H.F.; Han, X. Separation and preconcentration of MnVII/MnII speciation on crosslinked chitosan and determination by flame atomic absorption spectrometry. *Analyst* **2001**, *126*, 239–241.

73. Rakhtshah, J.; Shirkhanloo, H.; Mobarake, M.D. Simultaneously speciation and determination of manganese(II) and (VII) ions in water, food, and vegetable samples based on immobilization of N-acetylcysteine on multi-walled carbon nanotubes. *Food Chem.* **2022**, *389*, 133124.
